# A MYC-ZNF148-ID1/3 regulatory axis modulating cancer stem cell traits in aggressive breast cancer

**DOI:** 10.1038/s41389-022-00435-1

**Published:** 2022-10-07

**Authors:** Mijeong Kim, Manjot Singh, Bum-Kyu Lee, Moira Hibbs, Kirsty Richardson, Lesley Ellies, Larissa Wintle, Lisa M. Stuart, Jenny Y. Wang, Dominic C. Voon, Pilar Blancafort, Jianlong Wang, Jonghwan Kim, Peter J. Leedman, Andrew J. Woo

**Affiliations:** 1 Department of Molecular Biosciences, The University of Texas at Austin, Austin, TX 78712 USA; 2 Harry Perkins Institute of Medical Research, QEII Medical Centre, Nedlands and Centre for Medical Research, The University of Western Australia, Perth, WA 6000 Australia; 3 Centre for Precision Health, Edith Cowan University, Joondalup, WA 6027 Australia; 4 Department of Biomedical Sciences, Cancer Research Center, University at Albany, State University of New York, Rensselaer, NY 12144 USA; 5 RPH Research Centre, Royal Perth Hospital, Perth, WA 6000 Australia; 6 Division of Pharmacology and Toxicology, School of Biomedical Sciences, The University of Western Australia, Perth, WA 6000 Australia; 7 School of Medical Sciences, Faculty of Medicine and Health, University of Sydney, Sydney, NSW 2006 Australia; 8 Institute for Frontier Science Initiative, Kanazawa University, Kanazawa, 920-1192 Japan; 9 Cancer Research Institute, Kanazawa University, Kanazawa, 920-1192 Japan; 10 School of Human Sciences, The University of Western Australia, Perth, WA 6000 Australia; 11 The Greehey Children’s Cancer Research Institute, The University of Texas Health Science Center at San Antonio, San Antonio, TX 78229 USA; 12 Department of Medicine, Columbia Center for Human Development, Columbia Stem Cell Initiative, Herbert Irving Comprehensive Cancer Center, Columbia University Irving Medical Center, New York, NY 10032 USA; 13 School of Medical and Health Sciences, Edith Cowan University, Perth, WA 6000 Australia

**Keywords:** Breast cancer, Cancer genetics

## Abstract

The MYC proto-oncogene (*MYC*) is one of the most frequently overexpressed genes in breast cancer that drives cancer stem cell-like traits, resulting in aggressive disease progression and poor prognosis. In this study, we identified zinc finger transcription factor 148 (*ZNF148*, also called Zfp148 and ZBP-89) as a direct target of MYC. ZNF148 suppressed cell proliferation and migration and was transcriptionally repressed by MYC in breast cancer. Depletion of ZNF148 by short hairpin RNA (shRNA) and CRISPR/Cas9 increased triple-negative breast cancer (TNBC) cell proliferation and migration. Global transcriptome and chromatin occupancy analyses of ZNF148 revealed a central role in inhibiting cancer cell de-differentiation and migration. Mechanistically, we identified the Inhibitor of DNA binding 1 and 3 (*ID1, ID3*), drivers of cancer stemness and plasticity, as previously uncharacterized targets of transcriptional repression by ZNF148. Silencing of ZNF148 increased the stemness and tumorigenicity in TNBC cells. These findings uncover a previously unknown tumor suppressor role for ZNF148, and a transcriptional regulatory circuitry encompassing MYC, ZNF148, and ID1/3 in driving cancer stem cell traits in aggressive breast cancer.

## Introduction

Tumor initiation and progression often involve the dysregulation of developmentally important genes in cancer cells, endowing them with stem cell-like features such as enhanced self-renewal, invasiveness, and an aberrant differentiation state [1]. Triple-negative breast cancer (TNBC) cells that lack the expression of estrogen receptor (ER), progesterone receptor (PR), and human epidermal growth factor receptor 2 (HER2) [2], often exhibit the molecular and functional traits of cancer stem cells [3–9]. Despite advances in breast cancer therapy, the survival of TNBC patients remains poor, due to aggressive cancer progression, therapy resistance, metastasis, and recurrence, likely due to increased cancer stemness [10]. Consequently, in order to develop effective targeted therapy, there is a pressing need to identify all molecular regulators that define cancer stem cell traits in TNBC.

The MYC proto-oncogene is a transcription factor essential in stem/progenitor cell maintenance and differentiation [11]. *MYC* is frequently overexpressed in many cancers, and drives a cancer stem cell phenotype with enhanced cell growth, metastasis, and metabolic reprogramming [11–13]. In breast carcinomas, *MYC* is one of the most frequently dysregulated oncogenes, particularly in *BRCA1-*mutated, basal-like TNBCs, where *MYC* amplification is reported in up to 50% of cases [14–16]. In these cancers, the *BRCA1* gene acts as a tumor suppressor to repress the expansion of basal stem cells and basal-like breast cancers [17–19].

We previously reported a MYC-centered regulatory network in mouse embryonic stem (ES) cells that accounts for the similarity between the ES cells and cancer cells, and is associated with a worse prognosis in cancer, including cancers of the breast [20]. We found zinc finger protein 148 (ZNF148, also called Zfp148 and ZBP-89), a ubiquitously expressed Krüppel-like zinc finger protein, as a target of MYC-regulatory network in mouse ES cells [20]. Partial deletion of the ZNF148 locus causes defective primordial germ cell development in mice [21], though a complete deletion in the C57BL/6 mouse strain leads to postnatal lethality [22]. In malignant tumors, ZNF148 has been reported to be a suppressive [23–25] or oncogenic [26–28] factor, depending on the tumor cell of origins and tissue types. Despite ZNF148 being an established node of the MYC-network in ES cells, and the significance of MYC in driving cancer stem cells traits, the role of ZNF148 in breast cancer remains elusive.

The Inhibitor of DNA binding 3 (*ID3*) is a member of the ID protein family, consisting of four members (*ID1* to *ID4*). *ID1* and *ID3* share the closest sequence homology, and they are functionally redundant [29, 30]. Developmentally, it is well established that expression of the ID members is strongest in stem/progenitor cells, while their levels decrease upon differentiation. However, their expression frequently becomes reactivated in cancer, providing cancer cells with stem cell-like traits [31, 32]. In TNBC, ID1 and ID3 are requisite for self-renewal, metastasis, tumor re-initiation, and colonization, making them attractive targets for cancer stem cell therapy [30, 33–35].

Herein, we provide evidence for a direct transcriptional circuitry that functionally incorporates MYC, ZNF148, and ID1/3, regulating cancer stem cell traits in breast cancer. MYC actively represses ZNF148 expression, and in turn, the depletion of ZNF148 leads to de-repression of ID1/3, which drives the cancer stem cell phenotype. Thus, ZNF148 functions as a tumor suppressor, promoting the differentiated state of breast cancer cells, and suppressing cell proliferation, metastasis, and biosynthetic programs associated with cancer stem cells.

## Materials and methods

### Cell culture and In vitro clonogenic assay

MCF7, HCC1806, BT549 and MDA-MB-231 cells were cultured in RPMI-1640 (without phenol red) supplemented with 10% FCS, 2 mM Glutamine and 2% penicillin-streptomycin (P/S). HEK293T cells were cultured in DMEM High Glucose with 10% FCS and 2% P/S. MCF10A cells were cultured in DMEM/F12 supplemented with 5% horse serum, 20 ng/ml EGF, 100 ng/ml cholera toxin, 0.01 mg/ml insulin and 0.5 mg/ml hydrocortisone. All cell lines are sourced from ATCC and tested for mycoplasma contamination. Cells were maintained at 20–80% confluence at 37 °C with 5% CO_2_ air atmosphere. Clonogenic assays were performed as previously described [36]. In brief, 1 × 10^3^ MDA-MB-231 cells were seeded into 100 mm dishes and cultured for 12 days before cells were fixed with 100% methanol for 10 min and stained with 0.5% (w/v) crystal violet in 25% methanol.

### Expression constructs and oligonucleotides

The human ZNF148 cDNA was amplified from MGC clone 4423572 (GE Healthcare Dharmacon, Inc.) and cloned into the pEF-Biotag vector [37] for biotinylation-mediated chromatin immunoprecipitation followed by sequencing (BioChIP-seq). The cDNA was cloned into the pLeGO-iG2 vector [38] (a gift from Boris Fehse, Addgene plasmid # 27341), for transient transfection and stable lentivirus transduction. Oligonucleotides used for polymerase chain reactions (PCR) are listed in Supplemental Table 1.

### Western blotting

The ZNF148-N14 rabbit polyclonal antibody was raised as previously reported [37]. Antibodies specific for MYC (9402, Cell Signaling) and GAPDH (sc-25778, Santa Cruz Biotechnology Inc.) were purchased commercially. Western blotting (WB) was performed as described [37] using 10–25 µg of nuclear protein in each lane of 4–12% Bis-Tris gels (Invitrogen). All chemicals were purchased from Merk/Sigma-Aldrich, unless noted otherwise.

### Animal studies

All mammary orthotopic xenograft experiments were conducted in six to eight-week-old female NOD.Cg-Prkdc^*scid*^ ll2rg^tm1Wjl^/SzJ/Arc (NSG^TM^) mice (Animal Resources Center, WA, Australia). To generate mammary tumors in mice, 0.5 × 10^6^ MDA-MB-231-Luc cells stably transduced with empty vector control (EV) or ZNF148 cDNA (ZNF148^OE^), were resuspended in 2 mg/ml Matrigel™-HC (BD BioSciences) and injected into inguinal mammary fat pads of mice. To visualize the xenograft tumors in vivo, D-Luciferin (14681, Cayman Chemical) was injected intra-peritoneally (i.p.) into mice at 150 mg/kg of body weight, 12 min before the live imaging. To minimize the animal use, we performed power analysis using repeated-measures ANOVA with an F test for between-within subjects with Greenhouse-Geisser correction. Five mice per group provided sufficient power (>0.8) to detect an effect size of one. For imaging, randomized mice were placed under isoflurane induced anesthesia and orientated ventral side down to facilitate oxygen and isoflurane flow through the nose cones. Images were taken using IVIS Lumina II multispectral imaging system and analyzed with Living Image 4.2 Software (Caliper LS, Hopkinton, MA, USA). Xenograft experiments were not performed in a blinded manner. All experiments involving mice were approved by the Animal Ethics Committee at Harry Perkins Institute of Medical Research (Perth, WA, Australia).

### Biotinylation-mediated chromatin immunoprecipitation (BioChIP)

BioChIP was performed broadly as previously described [22]. MCF7 and MDA-MB-231 cells were grown to 60–80% confluence and cross-linked with 1% formaldehyde (methanol-free) for 10 min at room temperature and quenched with 125 mM glycine. The cells were washed and sequentially lysed in PIPES and SDS lysis buffer. Genomic DNA was sonicated at 4 °C with a Covaris m220 sonicator to 200 to 1000 base pair (bp) for quantitative ChIP-PCR and 200–400 bp for BioChIP-seq. Post immunoprecipitation, biotin-protein-DNA complexes were recovered with Dynabeads™ M-280 Streptavidin (11205D, Thermo Fisher Scientific), before washing and de-crosslinking and DNA purification for subsequent analyses.

### Cellular proliferation, viability, cell cycle, and scratch wound closure assays

MTT cell proliferation and viability assays (Roche) were performed as per the manufacturer’s instructions. In brief, breast cancer cells were seeded into 96-well plates at a density of 2 × 10^3^ cells/well, cultured for 96 h before being processed for MTT assay. Cell cycle analyses were performed on an Accuri^TM^ flow cytometer (BD Biosciences) using the BrdU kit (BD Biosciences) in accordance with the manufacturer’s protocol. For cell proliferation assays using IncuCyte® live-cell imaging (Essen BioScience), 6 × 10^3^ MDA-MB-231 and 4 × 10^3^ BT549 cells were seeded into 96-well plates and cultured for 100 h. For scratch wound closure assays, the cells were cultured in IncuCyte® ImageLock 96-well plates until a monolayer formed. The cells were serum starved 24 h prior to creating the scratch on the cell monolayer using WoundMaker (Essen BioScience). All images and confluence measurements were performed on an IncuCyte® live-cell imager and processed with the IncuCyte Zoom software (Essen BioScience).

### Quantitative RT-PCR and ChIP-PCR

RNA samples were prepared using RNeasy Mini Kit (QIAGEN) or SV Total RNA Isolation system (Promega). QuantiTect Reverse Transcription Kit (QIAGEN) was used to synthesize cDNA. Eluates from ChIP were purified using UltraPure™ Phenol:Chloroform:Isoamyl Alcohol (25:24:1, v/v) (Invitrogen™) and ethanol precipitated using a standard protocol. Quantitative PCR was performed on a Rotor-Gene 6000 thermocycler (Qiagen) using SensiMixPlus SYBR (Bioline). The expression levels of genes quantified by RT-PCR (RT-qPCR) are presented relative to that of GAPDH; while the enrichments of regulatory regions assayed by quantitative ChIP-PCR (ChIP-qPCR) were calculated relative to an unrelated region in exon 6 of the *ACTB* locus, as previously described [20, 22, 39].

### Transfection, RNA interference, CRISPR/Cas9, and virus transduction

Transfection of plasmid vectors was performed using FuGENE® HD (Promega) and Lipofectamine® 2000 transfection reagent (Thermo Fisher Scientific) as per the manufacturer’s protocol. Stable transduction of lentiviral short hairpin RNA (shRNA) knockdown in breast cancer cells was performed as previously described [22]. Short hairpin RNAs (shRNAs) seed sequences against MYC and ZNF148 are shown in Supplemental Table 2. Retroviral transduction of pMXs-GFP and pMXs-cMYC (human) vectors was performed essentially as previously described [37] using a pCL-10A1 packaging vector (Novus Biologicals). The CRISPR/Cas9 gRNA plasmid targeting exon 4 of ZNF148 (Target ID: HS0000451815) was purchased from Sigma-Aldrich.

### ALDEFLUOR assay

The Aldehyde dehydrogenase (ALDH) activity in MDA-MB-231 cells was measured using ALDEFLUOR^TM^ kit (01700, STEMCELL technologies), following the manufacturer’s protocol. In brief, 5 × 10^5^ cells were subjected to the ALDEFLUOR assay with 2.5 μL of activated ALDEFLUOR^TM^ reagent in the presence or absence of 5 μL of diethylaminobenzaldehyde (DEAB). Flow cytometry analysis was performed using Accuri^TM^ instruments (BD Biosciences).

### Sequencing analysis

RNA-sequencing (RNA-seq) was performed on Illumina HiSeq2000 system, and the sequenced reads were aligned to the human (GRCh38) genome using Salmon (v.0.7.2) [40]. Differentially expressed genes (DEGs) were determined using DEseq2 package. BioChIP-seq was performed on NextSeq 500 and the sequenced read were mapped to human (GRCh38) genome using Bowtie2 (v 0.5.9-r16) [41]. Peaks were called using MACS2 with a *q*-value cut off of 0.01 (v2.1.1) [42]. DNA binding motif analysis was performed using HOMER [43]. Binding and Expression Target Analysis (BETA) [44] was used to integrate and analyze BioChIP-seq and DEG data. GREAT (v4.0.4) [45], ShinyGO (v0.61) [46] and BETA were used for gene ontology functional annotation analysis.

### Statistical analyses

Student’s *t*-test (two-tailed) and Two-way ANOVA (ordinary) statistical analyses were performed using GraphPad Prism. Replicate experiments, sample size (*n*), mean values, SEM, SD, or CI error bars are indicated in the figure legends.

## Results

### ZNF148 is repressed by MYC in breast cancer

**Fig. 1:**
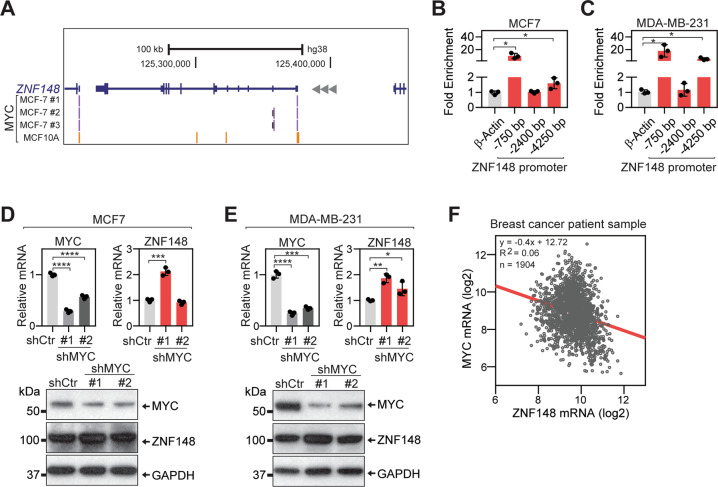
MYC represses ZNF148 expression by direct chromatin occupancy. **A** Schematic representation of ZNF148 locus on chromosome 3q21 reverse strand. Peak calls for MYC in MCF7 (purple bars), and MCF10A (yellow bars) cells from ENCODE [47] are indicated. **B** Quantitative ChIP-PCR (ChIP-qPCR) validation of MYC occupancy at −750, −2400, and −4250 bp upstream of ZNF148 promoter region in MCF7 cells (*n* = 3). **C** As in “**B**” for MDA-MB-231 cells. **D** Analysis of MYC and ZNF148 mRNA and protein in MCF7 cells transduced with the short-hairpin (sh) luciferase control (Ctr) and shMYC #1 and #2 lentiviruses, by RT-qPCR (top panel, *n* = 3) and Western blot (bottom panel). **E** As in “**C**” for MDA-MB-231 cells. **F** Scatter plot of MYC and ZNF148 mRNA levels (log2) from 1904 breast cancer patient samples in METABRIC cohort [56, 78]. Spearman correlations of −0.24 (*p* = 5.29^−27^) and Pearson correlations of −0.25 (*p* = 1.90^−27^) indicate an inverse correlation of ZNF148 and MYC mRNA levels and is depicted with a linear trend line in “red”. Student’s t-test, *P < 0.05. **P < 0.01. ***P < 0.001. ****P < 0.0001. Error bars indicate mean ± SD.

To investigate if MYC transcriptionally regulates *ZNF148* in breast cancer, we first examined the ChIP-seq data in the Encyclopedia of DNA Elements (ENCODE) database [47, 48]. Confirming our previous observation in mouse ES cells [20], MYC occupancy was observed at the proximal promoter of *ZNF148* in both MCF7 (ER positive, luminal subtype) and MCF10A (normal-like) breast cancer cells (Fig. 1A), and this region harbored several canonical and non-canonical E-box sequences (Supplemental Fig. 1A). To validate and extend this observation in TNBC cells, we performed ChIP-qPCR for MYC using MCF7 and a metastatic TNBC cell line, MDA-MB-231. This confirmed a significant enrichment of MYC at −750 and −4250 bp upstream regions of the *ZNF148* locus (Fig. 1B, C).

Next, we performed a loss-of-function study using short hairpin RNAs (shRNAs) against MYC to understand the impact of MYC depletion on *ZNF148* gene regulation. Two different shRNAs significantly reduced both MYC mRNA and protein levels in MCF7 and MDA-MB-231 cells, compared to the control samples transduced with an shRNA targeting firefly luciferase (shCtr) (Fig. 1D, E). While the majority of MYC transcriptional regulation has been linked to gene activation [11, 12], silencing of MYC, surprisingly resulted in increased ZNF148 mRNA and protein expression (Fig. 1D, E). Analysis of publicly available ChIP-seq data revealed the simultaneous presence of H3K27me3 repressive and H3K4me3 active histone marks on the ZNF148 promoter, consistent with a bivalent epigenetic state, suggesting the ZNF148 gene is poised for repression and activation (Supplemental Fig. 1B). To ascertain if this negative relationship also occurred in clinical specimens, we queried the patient-derived gene expression data in the Molecular Taxonomy of Breast Cancer International Consortium (METABRIC) datasets. Indeed, an inverse correlation between MYC and ZNF148 mRNA levels in breast cancer patients was observed (Fig. 1F). Collectively, these results support a direct transcriptional role for MYC in suppressing the expression of ZNF148 in breast cancer through chromatin occupancy.

### ZNF148 suppresses breast cancer cell growth and migration

**Fig. 2:**
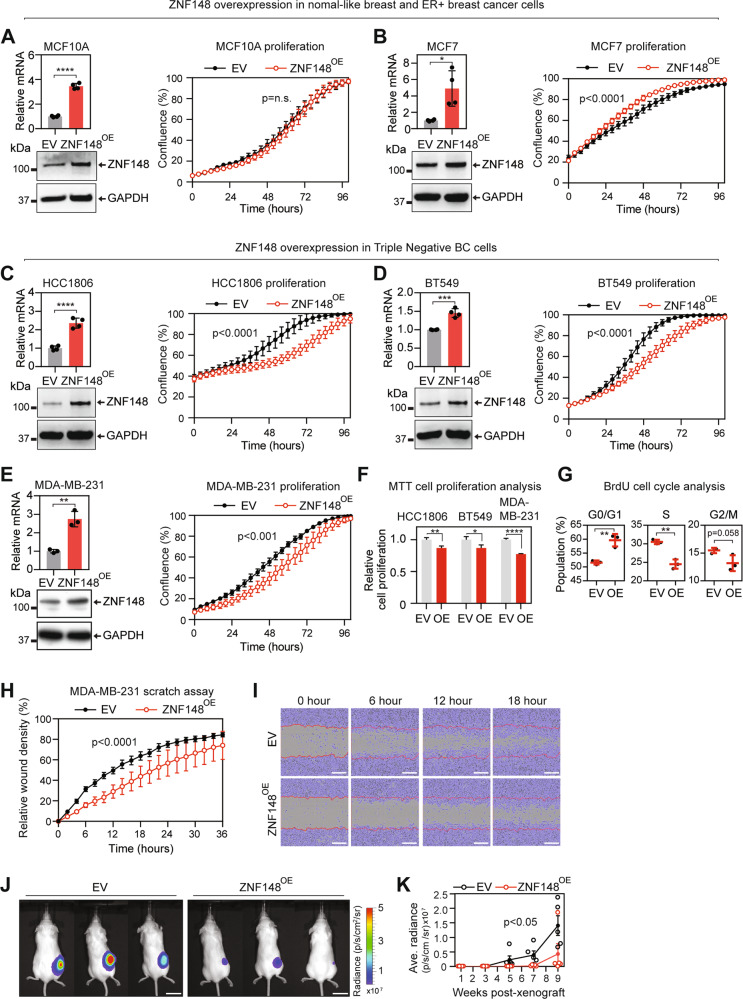
ZNF148 suppresses TNBC cancer cell growth and migration. **A** RT-qPCR analysis for ZNF148 mRNA transcripts, relative to GAPDH and empty vector (EV) control (*n* = 4, top panel), and Western blot analysis for proteins (bottom panel) in MCF10A cells transduced with ZNF148 cDNA (ZNF148^OE^) or empty vector (EV). Incucyte cell proliferation analysis (right panel) with percentage (%) area confluence on *y*-axis and time (hours) on *x*-axis, measured by IncuCyte® live-cell imaging over 96 h period (A representative plot from *n* = 3 biological replicates, each with *n* = 6 technical replicates, Two-way ANOVA *p*-values, Error bars indicate mean ± SD). **B**–**E** As in “**A**” for MCF7, HCC1806, BT549 and MDA-MB-231 (*n* = 3) cells respectively. **F** MTT cell proliferation analysis of cells in “**C**–**E**” 96 h post cell seeding (a representative graph from *n* = 3 biological replicates, each with *n* = 10 technical repeats, Student’s t-test, Error bars indicate mean ± SEM). **G** BrdU cell cycle analysis of MDA-MB-231-ZNF148^OE^ and the EV control cells (*n* = 3). **H** Scratch wound healing assay of cells in “**E**” using IncuCyte®. (*n* = 3, Two-way ANOVA *p*-value). **I** Representative images of the cells in “**E**” with the cell confluent area depicted in purple and the initial scratch front at time 0 depicted as a red line. Scale bars, 300 μM. **J** IVIS live animal imaging of NSG mice. Representative images NSG mice at 7 weeks posted engraftment of MDA-MB-231-Luciferase cells stably transduced with empty vector control (EV) or ZNF148 cDNA (ZNF148^OE^) (*n* = 5). **K** Line graph with dot plot showing biweekly average radiance values of five mice from week 1 to 9. Error bars indicate mean ± SEM. (*n* = 5, Two-way ANOVA *p*-value). Student’s t-test, *P < 0.05. **P < 0.01. ***P < 0.001. ****P < 0.0001.

To determine the functional significance of ZNF148 in breast cancer, we used lentivirus to stably express ectopic ZNF148 cDNA into ER+ breast cancer (MCF7), TNBC (MDA-MB-231, BT549, and HCC1806), and normal-like breast (MCF10A) cell lines. RT-qPCR and Western blot (WB) analysis confirmed the ectopic expression of ZNF148 in these cells (Fig. 2A–E). IncuCyte cell proliferation assay showed no change in the proliferation rate in MCF10A, but a marginal increase in ER + MCF7 cells that stably express ectopic ZNF148 (Fig. 2A, B). Interestingly, we observed a reduction in cell growth in all three TNBC cell lines that overexpress ZNF148 (Fig. 2C–F). BrdU cell cycle analysis of MDA-MB-231 cells expressing ectopic ZNF148 showed a significant reduction in the synthesis (S) phase as well as increased gap (G0/G1) phases compared to the empty vector (EV) control cells, confirming the anti-proliferative effect of ZNF148 (Fig. 2G). Subsequently, the impact of ZNF148 on cell migration in TNBC cell lines was investigated by the IncuCyte scratch assay. To account for ZNF148’s anti-proliferative effect, both EV controls and the ZNF148^OE^ cells were serum-starved for 24 h for cell cycle synchronization prior to the migration assay. Under this synchronized state, MDA-MB-231-ZNF148^OE^, BT549-ZNF148^OE^, and HCC1806-ZNF148^OE^ showed significantly slower wound closure compared with the control (Fig. 2H, I and Supplemental Fig. 2).

The above in vitro cellular assays indicate that ZNF148 exhibits classical tumor-suppressive traits with anti-proliferative and anti-migratory effects on TNBC cells. To validate this in vivo, we established firefly luciferase lentiviral expressing variants of EV control (MDA-MB-231-Luc-EV) and ZNF148-overexpressing (MDA-MB-231-Luc-ZNF148^OE^) cells. The established cells were xenografted into the 4th inguinal mammary fat pads of immunocompromised NSG^TM^ mice, and tumor growth was monitored by IVIS bioluminescence imaging. Consistent with the in vitro observations, the ZNF148-overexpressing MDA-MB-231-Luc-ZNF148^OE^ cells showed significantly reduced tumor growth in mice compared to the controls (Fig. 2J, K). Collectively, these data support a tumor suppressive role for ZNF148 in TNBC cells.

### Depletion of ZNF148 promotes breast cancer cell growth and migration

**Fig. 3:**
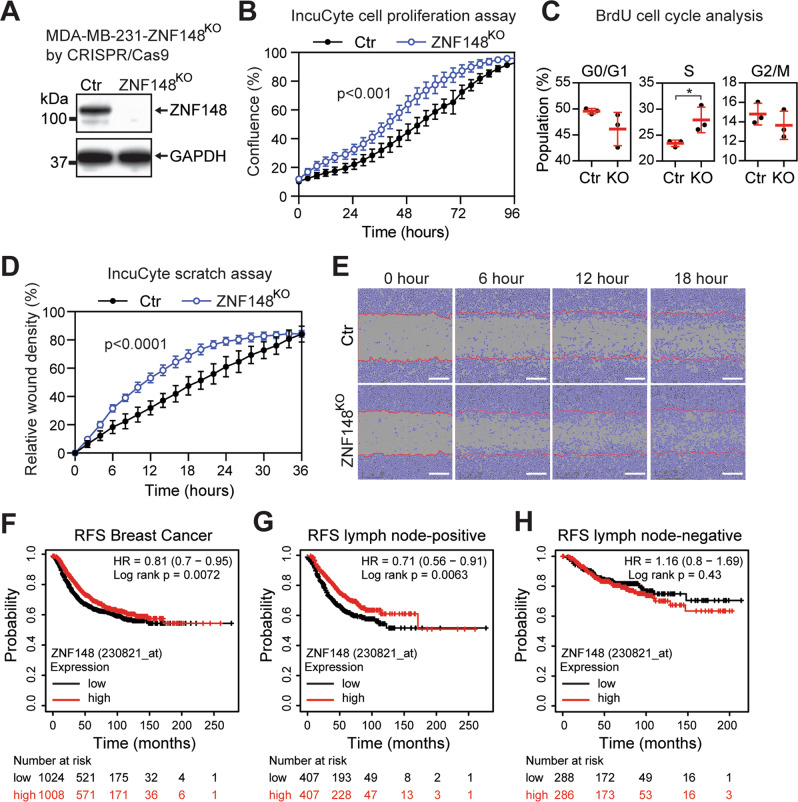
ZNF148 depletion promotes breast cancer cell growth, migration, and a low-level associate with a worse prognosis. **A** Western blot analysis of CRISPR/Cas9 ZNF148 knockout (KO) clone, compared to the wild type control (Ctr). **B** Percentage (%) area confluence of cells in “A”, measured by IncuCyte® live-cell imaging over 96 h period (a representative plot from *n* = 3 biological replicates, each with *n* = 6 technical replicates, Two-way ANOVA p-value, Error bars represent mean ± SD). **C** BrdU cell cycle analysis of cells in “A” (*n* = 3, Error bars = mean ± SD). **D** IncuCyte® scratch wound healing assay of cells in “**A**” (a representative plot from *n* = 3 biological replicates, each with *n* = 6 technical replicates, Two-way ANOVA *p*-value, Error bars indicate mean ± SD, Two-way ANOVA *p* < 0.0001). **E** Representative photographs of the cells in “**A**” with the cell confluent area depicted in purple and the initial scratch front at time 0 depicted as a red line. Scale bars, 300 μM. **F** Relapse-free survival analysis of entire breast cancer patients in Kaplan–Meir plotter database [79]. **G** As in “**F**” for lymph node-positive breast cancer cases. **H** As “**F**” for lymph node-negative cases. Student’s t-test, *P < 0.05. RFS, relapse-free survival.

To investigate the functional outcome of ZNF148 depletion, CRISPR/Cas9 gRNA that targets exon 4 of *ZNF148* was used to generate a knockout clonal line MDA-MB-231-ZNF148^KO^ that has a complete absence of the endogenous ZNF148 protein (Fig. 3A). The ablation of ZNF148 significantly increased both cell proliferation and the percentage of cells in S phase compared to the parental control cells (Fig. 3B, C). To circumvent potential clonal and off-target effects of the CRISPR/Cas9 gene targeting, we complemented this assay with RNA interference of ZNF148 by lentivirus-mediated shRNA. As expected, silencing *ZNF148* increased the cellular proliferation of both MDA-MB-231 and BT549 cells (Supplemental Fig. 3). We next performed an IncuCyte scratch assay to investigate the role of ZNF148 in cell migration. Under the same aforementioned serum-starved condition, the scratch wounds closed significantly faster in MDA-MB-231-ZNF148^KO^ cell cultures than in control cultures (Fig. 3D, E), again suggesting a role for ZNF148 in inhibiting cell migratory behavior.

To determine the clinical relevance of the observed cell line data, we examined the relapse-free survival (RFS) of breast cancer patients based on the expression level of *ZNF148*. In all breast cancer subtypes, patients with a higher level of *ZNF148* had a better survival chance, consistent with the observed tumor suppressive and anti-migratory effect of *ZNF148* (Fig. 3F). Of particular note, improved RFS associated with high *ZNF148* expression was observed only in patients with lymph node-positive, but not in the lymph node-negative cases of breast cancer (Fig. 3G, H). These results suggest that ZNF148’s tumor suppressive role is more significant in metastatic disease, which is typically associated with the aggressive TNBC subtype.

### ZNF148 restricts cellular proliferation via a transcriptional and epigenetic mechanism

**Fig. 4:**
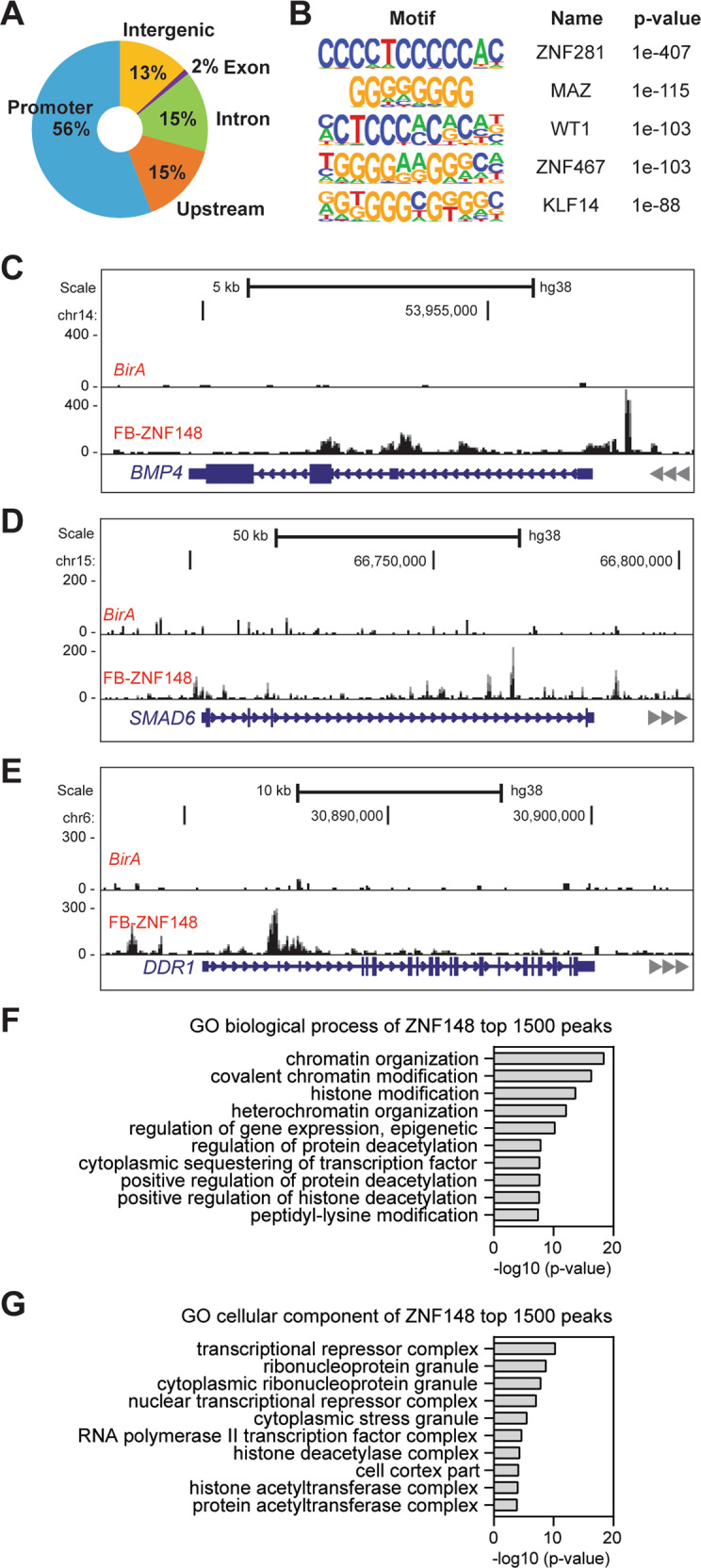
Genomic targets of ZNF148. **A** Doughnut chart showing the percentage distribution of ZNF148 chromatin occupancy peak locations determined by BioChIP-seq. **B** Consensus DNA binding motif analysis of ZNF148 occupied sequences (HOMER) [43]. The top 5 representative DNA binding sequence motifs were ranked by *p*-value and known transcription factors. **C** Representative BioChIP-seq signals at the bone morphogenetic protein 4 (*BMP4*) locus in MDA-MB-231 cells expressing *BirA* alone or *BirA* and FB-ZNF148. **D** As in “**C**” for SMAD family member 6 (*SMAD6*) locus. **E** As in “**C**” for discoidin domain receptor tyrosine kinase 1 (*DDR1*) locus. **F** GO biological process analysis of ZNF148 top 1500 peaks. **G** GO cellular component analysis of ZNF148 top 1500 peaks.

To understand the molecular basis for the tumor suppressor activities of ZNF148, we investigated the transcriptional targets of ZNF148. First, we introduced biotin-tagged ZNF148 into the parental MDA-MB-231-*BirA* control cells expressing biotin ligase *BirA*, and generated an MDA-MB-231-ZNF148^Bio^ cell line expressing metabolically biotinylated ZNF148 (Supplemental Fig. 4). We then employed the BioChIP-seq approach [49] to map the genome-wide chromatin occupancy of ZNF148. Our analysis revealed that ZNF148 occupied 12,991 genomic sites, with the majority (56%) of the occupancy at the promoter regions, 13% intergenic, 15% intron, and 15% in the upstream areas of genes (Fig. 4A). The ZNF148 consensus binding sequence motif was composed of consecutive cytosine (C) or guanine (G) repeats (Fig. 4B). The genome-wide occupancy pattern of ZNF148 in breast cancer was consistent with our previous observation in hematopoietic cells [22, 39]. Moreover, the consensus DNA binding sequence motif matched most significantly with another ZNF148 family member, ZNF281 (also called Zfp281 and ZBP-99) [50], consistent with our previous findings [22]. Some of the high scoring BioChIP-seq enrichment sites included genes that have been implicated in cancer metastases (*DDR1* [51], *BMP4* [52], and *SMAD6* [53]), and cell cycle and survival (*CDIP1* [54] and *PPP2R5A* [55]) (Fig. 4C–E and Supplemental Fig. 5).

Gene ontology (GO) biological process term analysis of the top 1500 ZNF148 target peaks revealed chromatin organization, covalent chromatin modification, histone modification, and heterochromatin organization to be the most significantly enriched biological processes (Fig. 4F). In the GO cellular component analysis, transcriptional repressor complex was the most enriched term (Fig. 4G). Collectively, these results suggest that ZNF148 mediates its tumor suppressor activities *via* a transcriptional and epigenetic mechanism to restrict cellular proliferation.

### ZNF148 regulates cell differentiation and migration

**Fig. 5:**
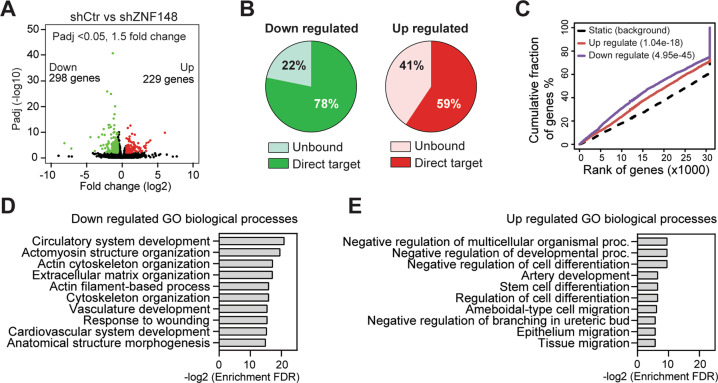
Transcriptome analysis of ZNF148 knockdown cells. **A** Volcano plot showing 229 upregulated (>1.5 fold) genes depicted in red and 298 downregulated (<1.5 fold) genes depicted in green (Padj < 0.05). **B** Pie charts showing the percentage of ZNF148-bound direct target genes or the unbound genes in up- or downregulated genes upon ZNF148 knockdown in MDA-MB-231 cells. **C** Transcription factor activating and repressive function prediction by BETA [44]. The upregulated (red line) and downregulated (purple line) ZNF148 target genes are cumulated by the rank on the basis of the regulatory potential score from high to low (*X*-axis). Both the up- or downregulated genes are significantly distributed above the non-differentially expressed static background (dashed line) by the Kolmogorov–Smirnov test [44]. **D** GO biological process analysis of downregulated genes from “**A**”. **E** As in “**D**” for the upregulated genes.

To further illustrate the genetic program regulated by ZNF148, we examined changes in the global transcriptomic landscape upon silencing ZNF148 in MDA-MB-231 cells by RNA-seq. To avoid potential clonal biases and allow the detection of immediate gene expression changes, RNA-seq was performed on total RNA isolated from early passage MDA-MB-231-shCtr and MDA-MB-231-shZNF148 cells. These analyses revealed 229 significantly upregulated genes (Padj < 0.05, Fold change > 1.5) and 298 downregulated genes (Padj < 0.05, Fold change < −1.5) upon the depletion of ZNF148 (Fig. 5A). We identified additional direct and indirect target genes of ZNF148 by combined analysis of BioChIP-seq and RNA-seq data. ZNF148 occupancy was identified in 78% of the downregulated genes, indicating that these genes are positively regulated by ZNF148. Similarly, 59% of the genes upregulated in MDA-MB-231-shZNF148 were the direct binding targets of ZNF148, identifying these as negatively regulated by ZNF148 (Fig. 5B). The plot of cumulative fraction of genes (%) versus rank of genes based on regulatory potential score (from high to low), predicted that ZNF148 functions as both an activator and repressor (Fig. 5C). GO term biological process analysis of the genes that are directly repressed by ZNF148 showed an enrichment for various macromolecule modification, biosynthesis, and metabolic processes, which are normally required for cell growth (Supplemental Fig. 6B). In contrast, genes directly activated by ZNF148 showed an enrichment for cellular component organization, organelle organization, inhibition of metabolic process, and anatomical structure morphogenesis, which are reminiscent of cellular differentiation processes (Supplemental Fig. 6A).

To gain further insight into the collective biological consequences of ZNF148 loss, including the direct and indirect changes, additional GO term analysis (biological processes) was performed on the 298 downregulated and 229 upregulated genes (Fig. 5D, E and Supplemental Tables 3, 4). While the downregulated genes were enriched in cytoskeleton and extracellular matrix organization-related terms, the upregulated genes were enriched in terms related to inhibiting development and differentiation (Fig. 5D, E). In line with the enhanced migration phenotype observed with the depletion of ZNF148 in vitro, we found enrichment of cell and tissue migration GO terms (Fig. 5E). These data collectively support the notion that ZNF148 is required for promoting the differentiation state and suppressing metabolic and migratory processes in breast cancer cells.

### Loss of ZNF148 enhances stemness of breast cancer cells

**Fig. 6:**
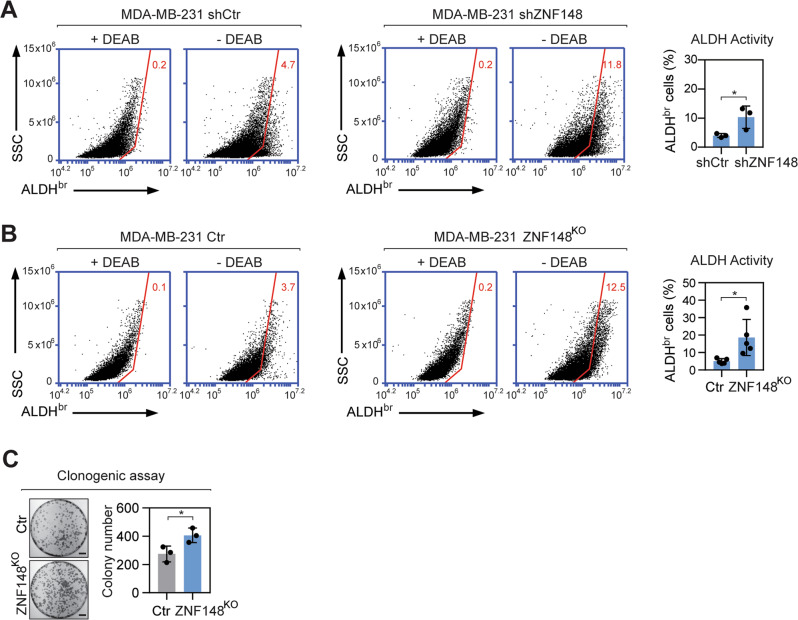
Loss of ZNF148 increases breast cancer cell stemness. **A** Representative flow cytometry plots and a bar graph, showing the percentage of viable Aldehyde dehydrogenase bright (ALDH^br^) shCtr and shZNF148 MDA-MB-231 cells in the presence (+) or absence (−) of diethylaminobenzaldehyde (DEAB) (*n* = 3). **B** As in “**A**” showing the percentage of ALDH^br^ cells in MDA-MB-231 Control and MDA-MB-231-ZNF148^KO^ cells (*n* = 5). **C** Representative image of clonogenic assay of cells in “**B**” stained with crystal violet (*n* = 3). Scale bar = 1 cm. Error bars represent mean ± SD. Student’s t-test, *P < 0.05. SSC side scatter; ALDH^br^ Aldehyde dehydrogenase bright; Ctr Control; KO knockout; DEAB diethylaminobenzaldehyde.

A high level of MYC drives stemness and an aggressive phenotype in breast cancer [11, 12, 14, 15, 56]. Based on our finding, we posit that MYC negatively regulates ZNF148 (Fig. 1) to repress biological processes involving cellular differentiation and development (Fig. 5), fostering cancer stem-cell-like features in breast cancer cells. To test our hypothesis, we measured aldehyde dehydrogenase (ALDH) enzymatic activity, which is elevated in cancer stem cell populations [4, 5, 9]. Upon silencing ZNF148 using shRNA, a significant increase in the percentage of ALDH bright (ALDH^br^) cells was observed in MDA-MB-231-shZNF148, compared with the basal level in MDA-MB-231-shCtr cells (Fig. 6A). Consistent with this, the percentage of ALDH^br^ cells in MDA-MB-231-ZNF148^KO^ cells likewise increased significantly compared to the control cells (Fig. 6B). In addition, clonogenic assays, which measure stem-like cell growth in vitro [57, 58], revealed increased colony formation in MDA-MB-231-ZNF148^KO^ cells compared to the control, further validating our hypothesis that ZNF148 is a negative regulator of stemness in breast cancer (Fig. 6C).

### ZNF148 transcriptionally represses ID1 and ID3

**Fig. 7:**
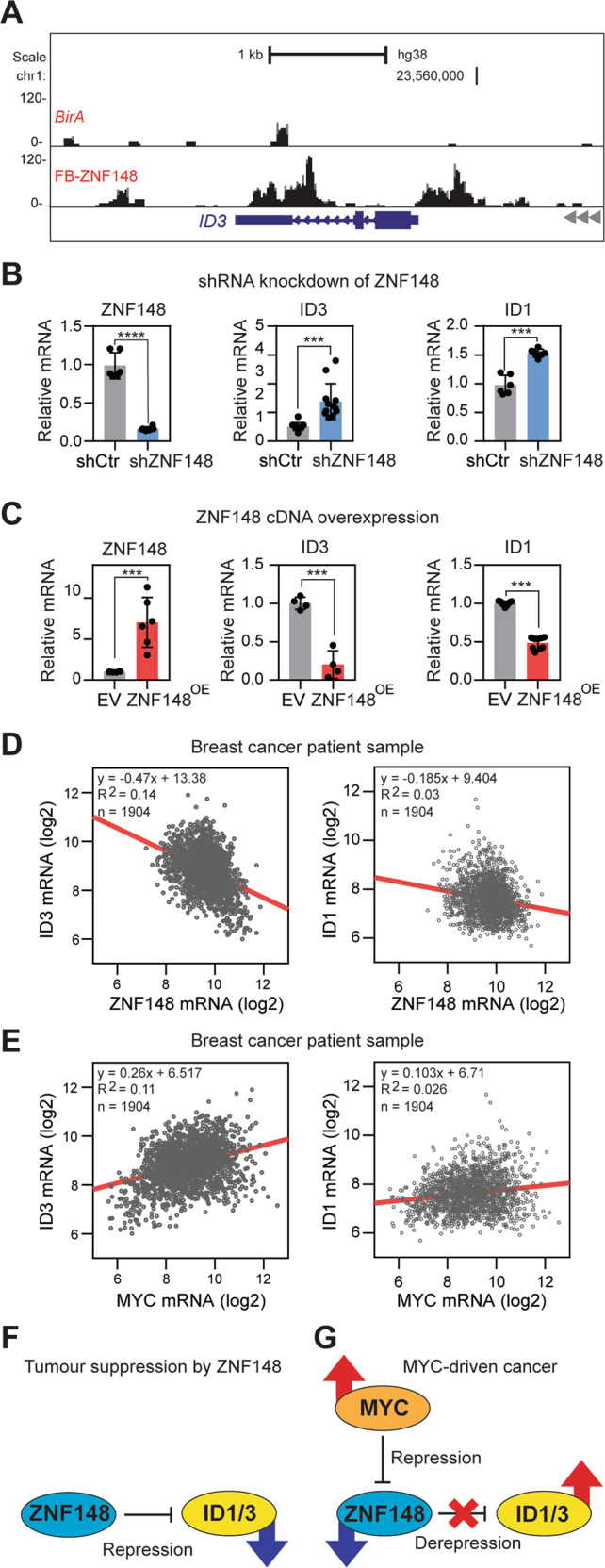
ZNF148 represses ID1/3. **A** BioChIP-seq signals at the inhibitor of DNA binding 3 (*ID3*) locus in MDA-MB-231 cells expressing *BirA* alone or *BirA* and FB-ZNF148. **B** RT-qPCR analysis for ZNF148 (left panel, *n* = 6), ID3 (middle panel, *n* = 12) and ID1 (right panel, *n* = 6) mRNA transcripts, relative to GAPDH and shControl (shCtr), in shZNF148 MDA-MB-231 cells. **C** RT-qPCR analysis for ZNF148 (left panel, *n* = 6), ID3 (middle panel, *n* = 4) and ID1 (right panel, *n* = 9) mRNA transcripts, relative to GAPDH and empty vector (EV) control, in ZNF148 overexpressing MDA-MB-231 cells. **D** Scatter plots of ID3 vs. ZNF148 (Pearson r = −0.3776, *p* < 0.0001), and ID1 vs. ZNF148 (Pearson r = −0.1761, *p* < 0.0001) mRNA levels (log2) from 1904 breast cancer patient samples in METABRIC cohort [56, 78]. **E** Scatter plot of ID3 vs MYC and ID1 vs MYC as in “D”, with Pearson r = 0.3359 (*p* < 0.0001) and Pearson r = 0.1590 (*p* < 0.0001) respectively, indicating a direct correlation of ID3/1 and MYC mRNA levels. **F** Schematic diagram of ZNF148 actively repressing ID1/3 gene expression. **G** Schematic diagram of regulatory circuitry between MYC, ZNF148, and ID1/3 in MYC-driven stem cell-like cancer. Student’s t-test, ***P < 0.001. ****P < 0.0001. Error bars indicate ± SD.

As ZNF148 suppresses stemness in MDA-MB-231 cells, we reasoned that certain target gene(s) of ZNF148 promoting stemness in cancer cells, are repressed by ZNF148. To identify downstream target gene(s) that enhance stem-like features in breast cancer, we ranked ZNF148 ChIP-enrichment scores, and the expression fold changes of genes within the enriched GO terms (Fig. 5E) and identified Inhibitor of DNA binding 3 HLH protein (*ID3*) as a potential candidate. ID1 and ID3 proteins are functionally redundant and have been shown to promote breast cancer cell self-renewal, tumor cell dissemination, and metastatic colonization of the lung and tumor re-initiation [33–35]. Importantly, the ZNF148 occupancy was significantly enriched at the promoter and gene body of ID3 (Fig. 7A). A closer examination of the other family members of ID genes, *ID1, ID2, and ID4* loci, all showed ZNF148 occupancy at the promoter and gene body, similar to ID3, albeit statistically less significant in MACS2 peak calling scores (Supplemental Fig. 7). Upon silencing of ZNF148 mRNA, we observed a dramatic increase of *ID3* and *ID1* transcription (Fig. 7B). Conversely, the overexpression of ZNF148 significantly decreased *ID3* and *ID1* (Fig. 7C). These data collectively provide compelling evidence that ZNF148 actively represses *ID1/3* genes in breast cancer cells.

To demonstrate the MYC-ZNF148-ID1/3 regulatory axis, we overexpressed MYC and GFP (control) cDNA in untransformed MCF10A breast epithelial cells using retroviruses and monitored the changes in *MYC*, *ZNF148*, and *ID1/3* transcripts. Consistent with the repressive role of MYC on *ZNF148*, the MYC overexpression reduced the *ZNF148* transcript level, subsequently conveying an increase in *ID3* expression (Supplemental Fig. 8A). Next, we used lentivirus-mediated shRNA transduction to downregulate ZNF148 in MCF10A-GFP and MCF10A-MYC^OE^ cells (Supplemental Fig. 8B). The ZNF148 depletion increased the *MYC* transcript level in MCF10A-GFP cells, consistent with the inverse expression correlation found in breast cancer patients (Supplemental Figs. 8B and 1F). Furthermore, the *ID3* expression increased with the ZNF148 depletion, or with the overexpression of MYC, while simultaneous depletion of ZNF148, and MYC overexpression led to an additive increase in *ID3* transcripts. For *ID1*, the gene expression changes did not follow that of *ID3* upon MYC overexpression and ZNF148 depletion, indicating an additional layer of regulatory mechanisms in MCF10A cells. Collectively, these data demonstrate that the genetic relationship between the MYC-ZNF148-ID3 axis is conserved across mammary epithelial cells irrespective of their transformed status.

Finally, we queried the METABRIC breast cancer patient data to examine the correlation between *MYC*, *ZNF148,* and *ID1/3* expression levels. Indeed, an inverse correlation between *ZNF148* and both *ID1/3* was observed (Fig. 7D). Furthermore, a direct proportional expression between *MYC* and *ID1/3* was observed (Fig. 7E), further strengthening the existence of a MYC, ZNF148, and ID1/3 regulatory axis in controlling the stemness in breast cancer (Fig. 7F, G).

## Discussion

Despite the importance of ID genes driving the cancer stem cell phenotype in a broad array of epithelial cancers, their regulatory mechanism remains incompletely understood. In this study, we uncover a novel role of ZNF148 in suppressing TNBC cell growth and metastasis and provide evidence for a direct regulatory circuitry between MYC, ZNF148, and ID1/3 that impacts stem cell traits in breast cancer. We show ID1 and ID3 are transcriptionally repressed targets of ZNF148, and that silencing of ZNF148 derepresses their expression, resulting in increased stemness of TNBC cells.

Patients with a higher level of ZNF148 show improved survival, but interestingly, the benefit of having a high ZNF148 gene dosage is accentuated only in metastatic, lymph-node positive disease (Fig. 3). It is possible that the effect of ZNF148 dosage in breast cancer is more pronounced in the context of MYC, stemness, and metastatic state of the cells. Our in vitro cell line data suggests that the anti-proliferative effect of ZNF148 is significant in TNBC but not in ER+ cells (Fig. 2). MDA-MB-231, in particular, is a highly metastatic TNBC cell line with pronounced MYC-driven cancer stem cell features [59, 60].

Oncogenic MYC alters global transcriptional programs to drive stem cell traits in cancer [11, 61, 62], while concomitantly downregulating tumor suppressor genes and cell cycle regulators [63–65]. Mechanistically, polycomb repressive complex 2 (PRC2) has been shown to interact with MYC proteins to induce transcriptional silencing of the target genes [66, 67]. SUZ12 and EZH2 are core subunits of PRC2; indeed, both were enriched at the promoter region of *ZNF148* (Supplementary Fig. 1B). SUZ12 and EZH2 occupancy, along with E-box motifs and MYC occupancy, and H3K27me3 marks at the regulatory region of the *ZNF148* locus provide a possible mechanism for MYC-induced repression of ZNF148 in breast cancer.

MYC also represses lineage-specific transcription factors in mammary tissues, such as *GATA3* and *ESR1* to drive cancer stem cell-like states in breast cancer [68]. We previously reported physical interaction between ZNF148 and GATA3 proteins and possible functional cooperation between them in hematopoietic cells [37]. It is possible that ZNF148 may cooperate with GATA3 to drive mammary tissue development, but without a mammary-specific mouse knockout study, whether ZNF148 also plays a lineage-specific transcriptional role remains unclear. Our current study supports a role for ZNF148 as a tumor suppressor gene in metastatic TNBC, that is actively repressed by MYC.

The level of MYC expression is exceptionally high in basal-like TNBCs that harbor *BRCA1* mutation [14–16]. The *BRCA1* gene suppressed basal stem cell expansion during mammary tumor development [17–19] and downregulated MYC expression [69], quenching the MYC-driven oncogenic pathways. Interestingly, the same study identified ZNF148 as a gene significantly upregulated by BRCA1 [69]. Although it was not clear whether BRCA1 directly contributed to transcriptional activation of ZNF148, the inversed gene expression pattern between ZNF148 and MYC is consistent with our findings. It is possible that the tumor suppression by BRCA1 may be in part mediated by ZNF148 activation as a result of MYC downregulation.

ZNF148 binds to GC-rich DNA motifs and shares the most similarity with ZNF281 (Fig. 4B). We recently reported similar GC-rich DNA consensus motifs, unique and overlapping chromatin occupancy sites for ZNF148 and ZNF281, and their functional redundancy in myelogenous leukemia cells [22]. Recently, ZNF281 was shown to activate epithelial to mesenchymal transition (EMT) transcription factors, ZEB1 and SNAI1 *via* activation of the TGF-β pathway, promoting EMT and metastasis in breast cancer [70]. Despite the similar DNA binding motifs between ZNF148 and ZNF281, we did not observe the chromatin occupancy of ZNF148 on ZEB1 or SNAI1 loci. Moreover, contrary to the ZNF281’s pro-metastatic role, ectopic expression of ZNF148 inhibited cell migration (Fig. 2H, I and Supplemental Fig. 2). ZNF281 is 80% homologous to ZNF148 in the DNA-binding domains, but only 3 to 40% similar in the N-terminal and C-terminal domains. These structural features may provide a foundation for shared chromatin occupancy but a divergent role at the occupied sites. There may be competition for occupancy at the same regulatory loci between ZNF148 and ZNF281, but mediate different functions by recruiting different cofactors, resulting in different net phenotypic outcomes.

The TGF-β pathway has a dual role acting as either tumor suppressive or oncogenic during carcinogenesis [71]. As a tumor suppressor, TGF-β reduces the breast cancer stem cell population, and cell proliferation *via MYC*, *ID1,* and *ID3* genes [72, 73]. Given the MYC-ZNF148-ID1/3 regulatory axis found in this study, ZNF148 may be involved in the TGF-β downstream pathways, mediating the repression of *ID1/3* genes, thus reducing the cancer stem cell properties in breast cancer. In the breast tumor microenvironment, TGF-β is oncogenic by priming cancer cells for metastasis to the lungs [74], facilitating the dissemination and colonization of cancer cells that have undergone EMT *via* ID1 mediated mesenchymal to epithelial transition (MET) [75]. Considering the recent finding that ZNF281 promotes metastasis *via* the TGF-β pathway, and an opposing role of ZNF148 involving *MYC* and *ID* genes, both ZNF148 and ZNF281 may be integral transcriptional regulators of the TGF-β pathway. Further work addressing both ZNF148 and ZNF281 within the same cellular context will be required to better understand the regulatory mechanisms contributing to the cancer stem cell state and metastatic progression.

In summary, our study adds ZNF148 to a cadre of tumor suppressors that MYC actively represses, particularly in breast cancer. Currently, various therapeutic approaches are being investigated to target both MYC and the ID proteins [12, 31, 76]. Our findings suggest that upregulating ZNF148 in breast cancer could also be beneficial. Sodium butyrate, an HDAC inhibitor was shown to increase ZNF148 expression in colorectal cancer cells [77], however, it did not change the ZNF148 levels in breast cancer cells (data not shown). Considering the ubiquitous nature of ZNF148 expression, the MYC, ZNF148, ID1/3 regulatory axis may be present in a broad array of cancers driven by MYC and ID proteins. Future work to identify therapeutic agents that upregulate ZNF148 could be of clinical importance, not only in breast cancer, but in various cancers with aggressive cancer stem cell-like traits.

## Supplementary information

Supplemental figures

Supplemental Table S1

Supplemental Table S2

Supplemental Table S3

Supplemental Table S4

## Data Availability

The RNA-seq and BioChIP-seq data generated in this study have been deposited in the Gene Expression Omnibus (GEO) public database under the accession numbers GSE132953 and GSE147020, respectively.
