# Supplementary material for: A MYC-ZNF148-ID1/3 regulatory axis modulating cancer stem cell traits in aggressive breast cancer
Source: Oncogenesis. 2022 Oct 7;11(1):60. doi: 10.1038/s41389-022-00435-1 (PMC9546828; doi:10.1038/s41389-022-00435-1)
Supplement: Supplementary file 1 — Supplemental figures [file 41389_2022_435_MOESM1_ESM.docx]

**SUPPLEMENTARY FIGURES**

| **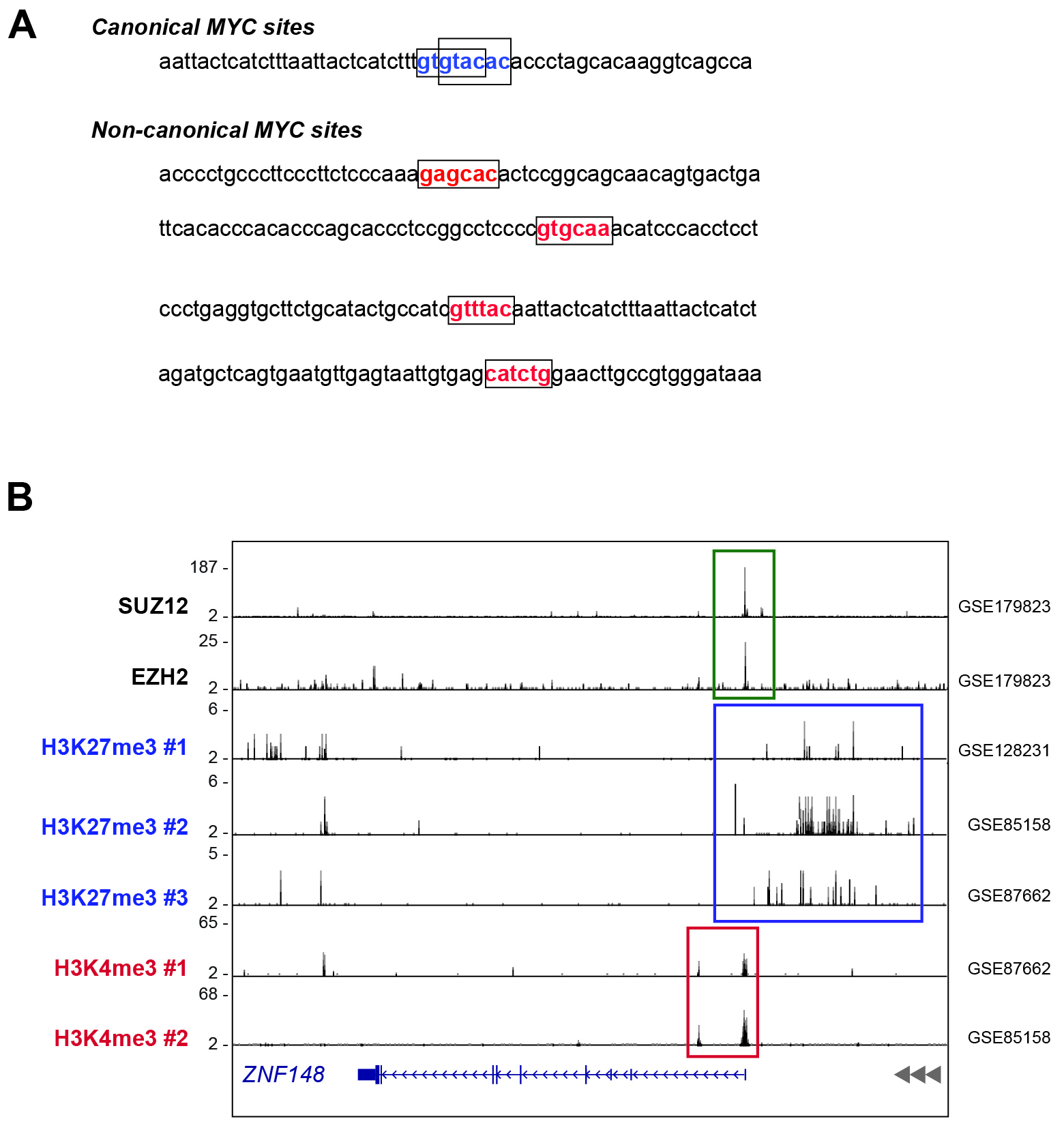** |
| --- |
| **Supplemental Figure 1. MYC occupancy and bivalent histone marks at the upstream region of *ZNF148*.** (A) Upstream DNA sequences of *ZNF148* near MYC ChIP-seq signal peaks in “Fig. 1A” containing canonical E-box sequences in “blue”. E-box sequence “CATGTG” or GTGTAC in reverse, or GTACAC complement sequence are boxed separately. Non-canonical E-box sequences are denoted in “red” and boxed accordingly. (B) Schematic representation of ZNF148 locus on chromosome 3q21 reverse strand and representative ChIP-seq signals for H3K27me3 #1-3 (GSE128231, GSE85158 and GSE87662), H3K4me3 #1-2 (GSE87662 and GSE85158), SUZ12 (GSE179823) and EZH2 (GSE179823). Rectangle boxes in “red”, “blue”, and “green” denote enriched ChIP-seq signals of H3K4me3, H3K27me3 and SUZ12/EZH2, respectively, at the upstream region of ZNF148 locus. |

| **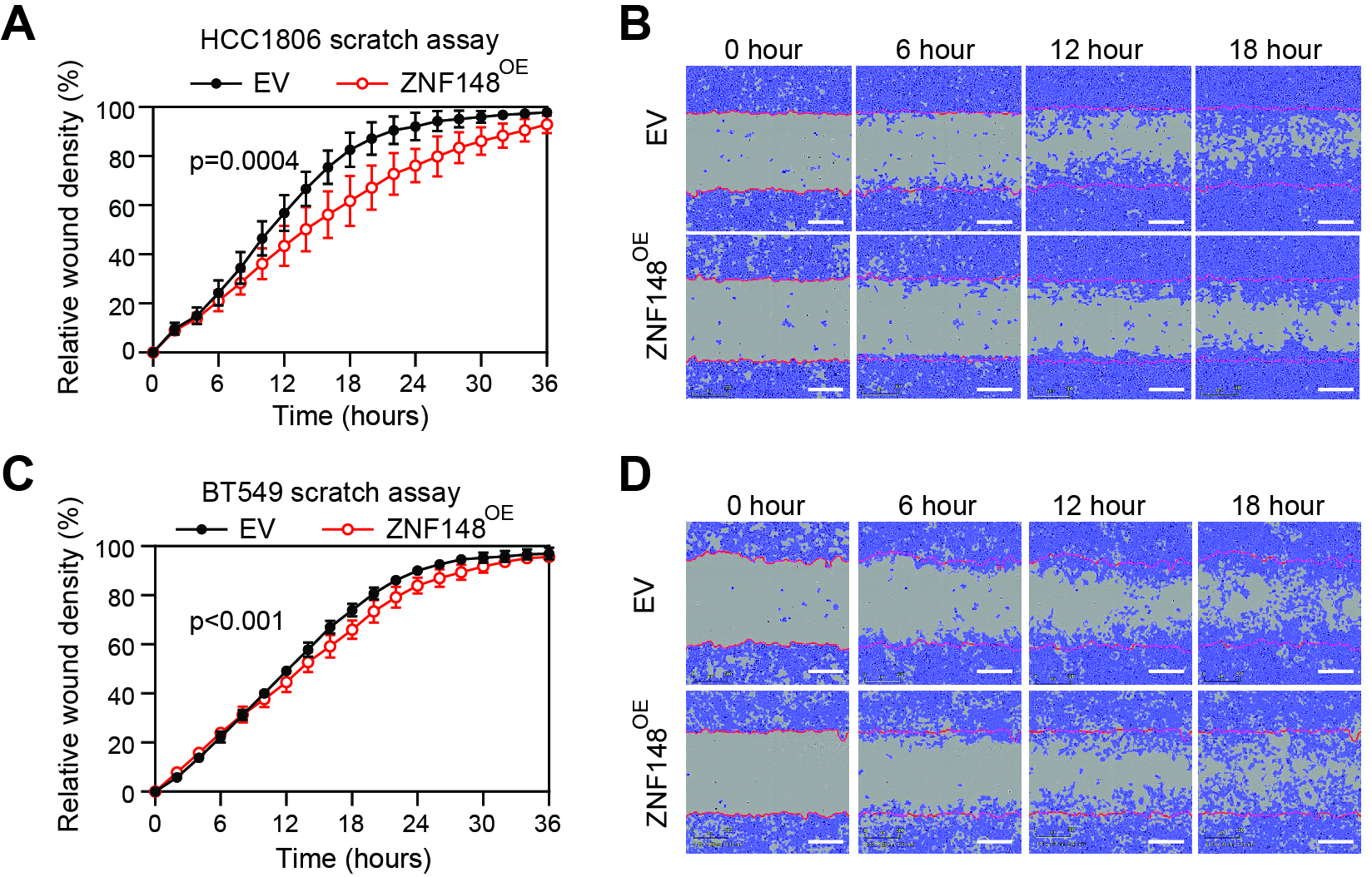** |
| --- |
| **Supplemental Figure 2.** ZNF148 suppresses TNBC cancer cell migration. (A) Scratch wound healing assay of HCC1806-EV and HCC1806-ZNF148^OE^ cells in “Fig. 2C” using IncuCyte®. (a representative plot from n = 3 biological replicates, each with n = 6 technical replicates, Two-way ANOVA p-value, Error bars indicate mean ± SD). (B) Representative images of the cells in “A” with the cell confluent area depicted in purple and the initial scratch front at time 0 depicted as a red line. Scale bars, 300 μM. (C) As in “A” for BT549-EV and BT549-ZNF148^OE^ cells in “Fig. 2D”. (D) Representative images of cells in “C”. |

| **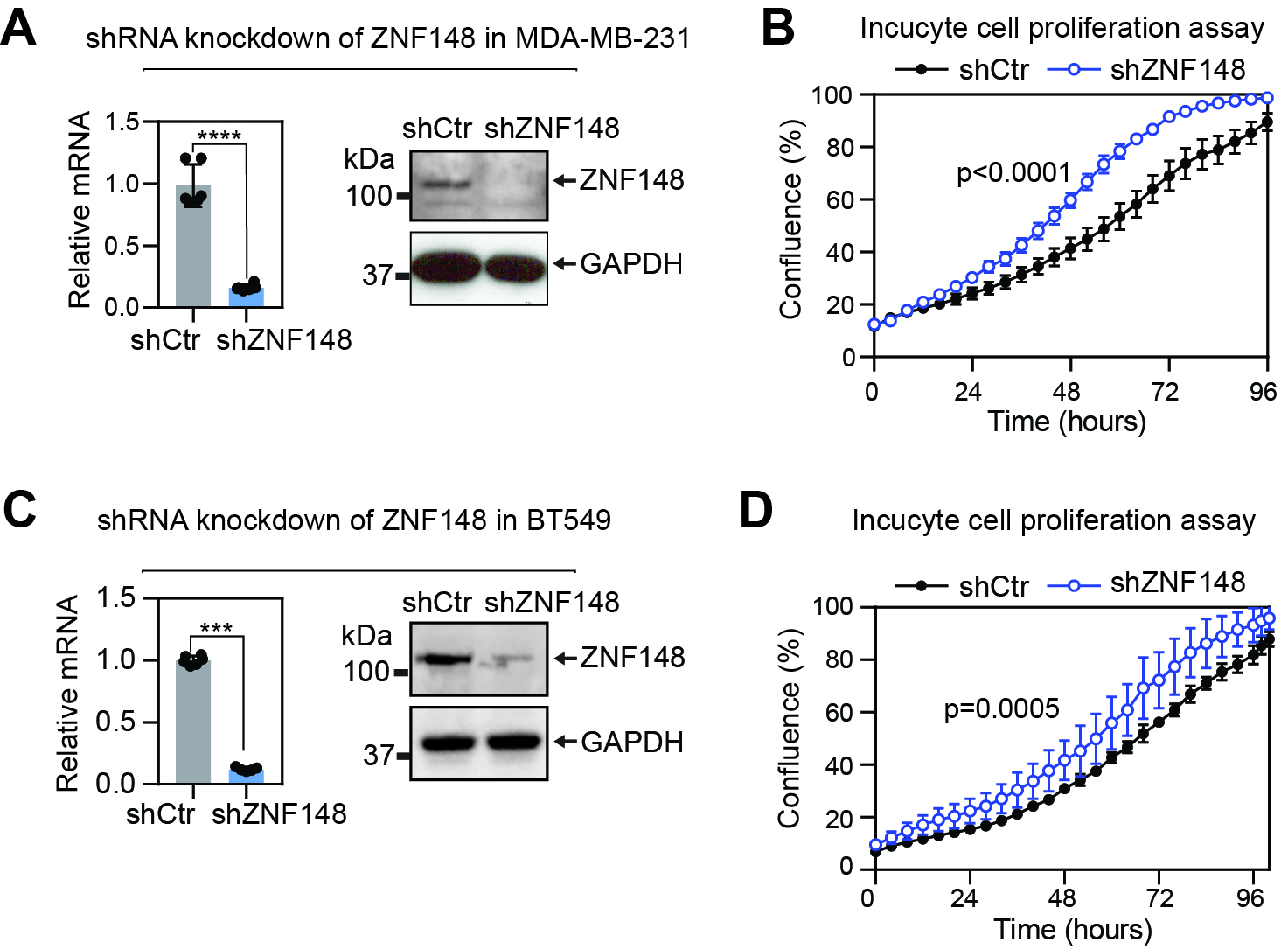** |
| --- |
| **Supplemental Figure 3.** ZNF148 depletion promotes breast cancer cell growth. (A) RT-qPCR (left panel, n = 6) and Western blot (right panel) analysis of ZNF148 levels in shZNF148 knockdown MDA-MB-231 cells compared to the shControl (shCtr). Error bars indicate ± SD. (B) Percentage (%) area confluence of cells in “A”, measured by IncuCyte® live-cell imaging over 96 hours period (a representative plot from n = 3 biological replicates, each with n = 6 technical replicates, Two-way ANOVA p-value, Error bars indicate mean ± SD). (C) As in “A” for shZNF148 in BT549 cells. (D) Percentage (%) area confluence of cells in “C”, measured by IncuCyte® live-cell imaging over 96 hours period (a representative plot from n = 3 biological replicates, each with n = 6 technical replicates, Two-way ANOVA p = 0.0005). Student's t-test, ***P* < 0.01. ****P* < 0.001. |

| **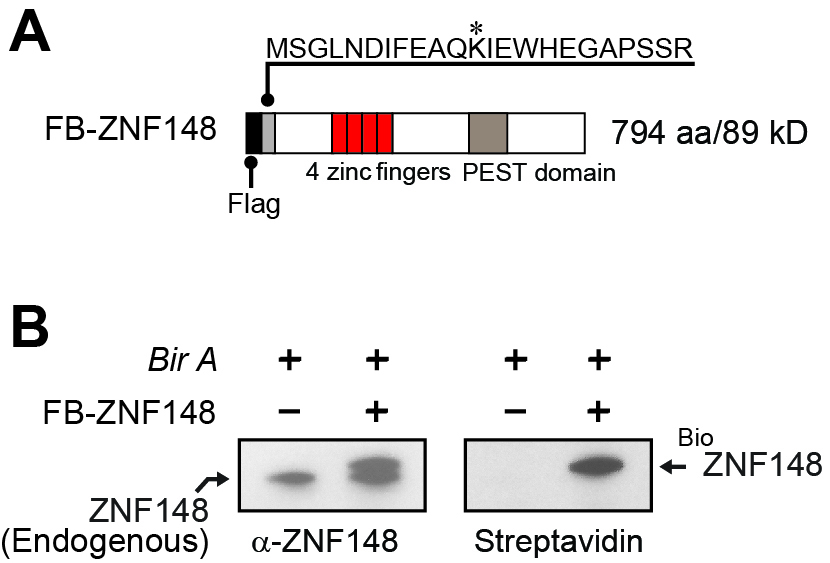** |
| --- |
| **Supplemental Figure 4.** Generation of MDA-MB-231 clones stably expressing ZNF148 flag-biotin (FB). (A) Schematic diagram of ZNF148 containing the amino-terminal FLAG peptide (black box) and the *BirA* recognition sequence (FLAG-Bio). The biotin acceptor lysine is indicated with an asterisk. (B) Western blot analysis of nuclear extracts from MDA-MB-231 clones stably expressing *BirA* alone or *BirA* and ZNF148^FB^. These blots are stripped and re-probed with streptavidin horseradish peroxidase as indicated. The ZNF148 protein bands are indicated by an arrow. Abbreviation: *BirA.*, E. coli biotin ligase. |

| 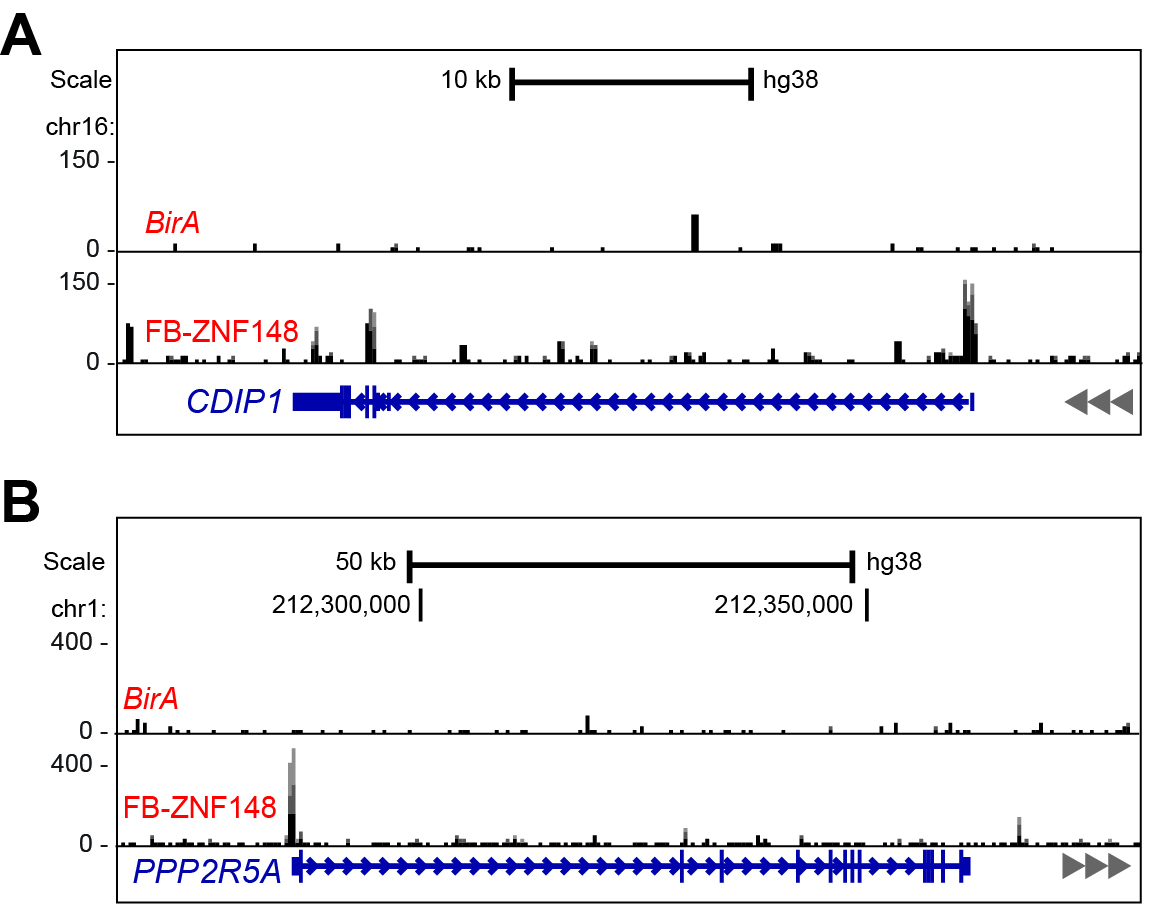 |
| --- |
| **Supplemental Figure 5.** Representative ZNF148 BioChIP-seq signals (A) BioChIP-seq signals at the cell death inducing p53 target 1 (*CDIP1*) locus in MDA-MB-231 cells expressing *BirA* alone or *BirA* and FB-ZNF148. (B) As in “A” for protein phosphatase 2 regulatory subunit B'alpha (*PPP2R5A*) locus. |

| **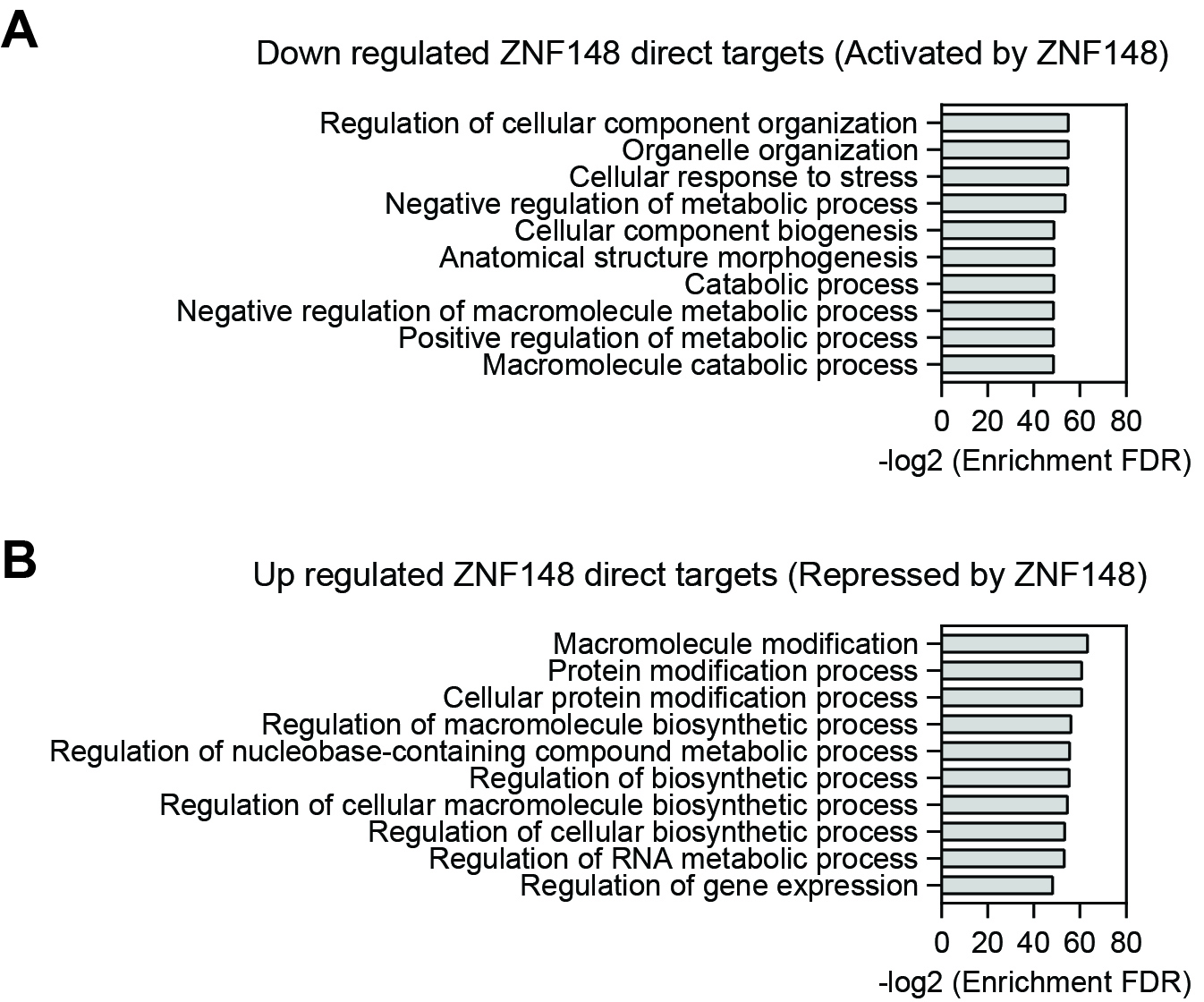** |
| --- |
| **Supplemental Figure 6.** GO term analysis of direct ZNF148 target genes changed in ZNF148 depleted MDA-MB-231 cells. (A) GO biological process analysis of direct ZNF148 target genes down-regulated with ZNF148 depletion *via* shRNA. (B) As in “A” for ZNF148 direct target genes up-regulated with ZNF148 depletion. |

| **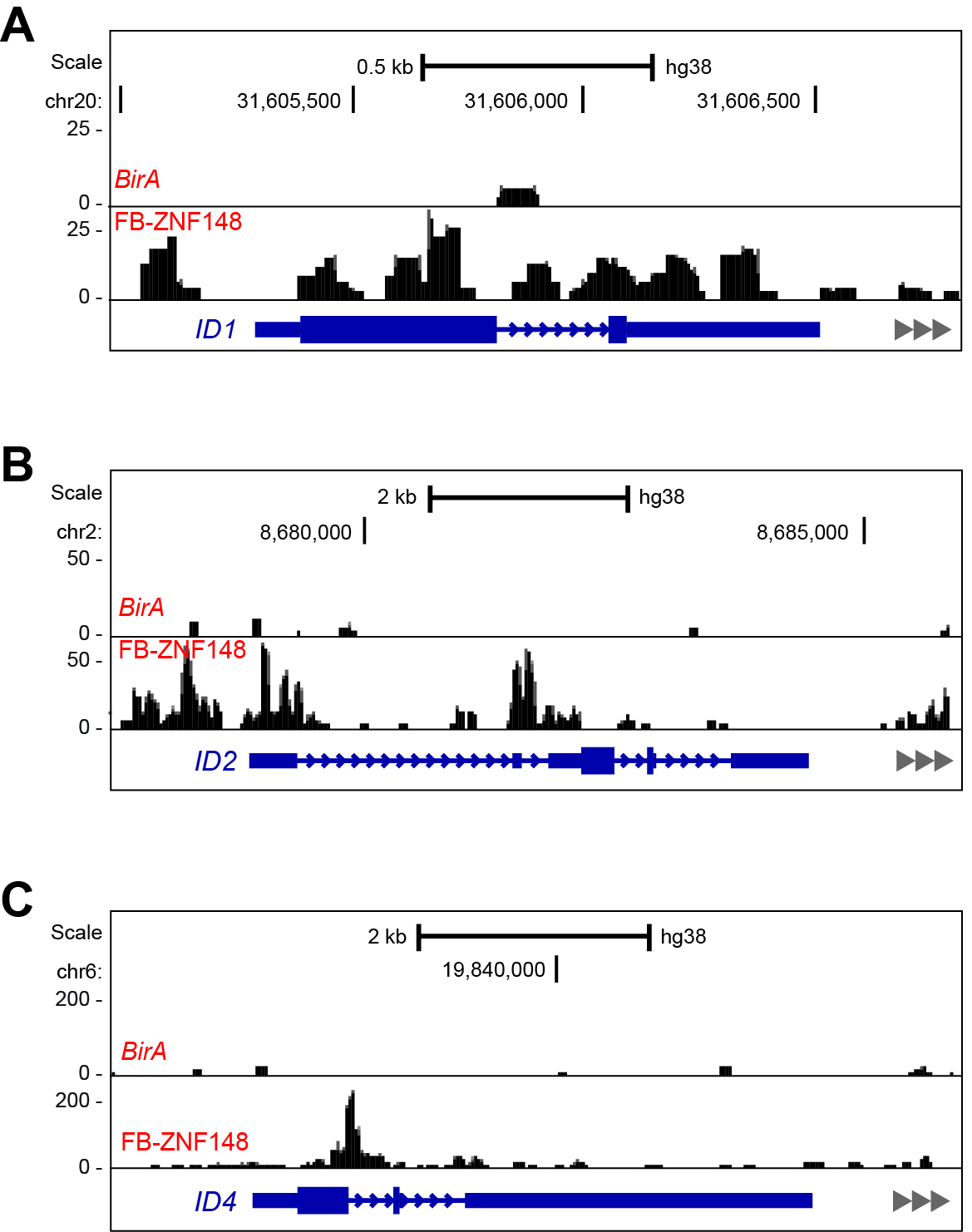** |
| --- |
| **Supplemental Figure 7.** Representative ZNF148 BioChIP-seq signals. (A) BioChIP-seq signals at the inhibitor of DNA binding 1 (*ID1*) locus in MDA-MB-231 cells expressing *BirA* alone or *BirA* and FB-ZNF148. (B) As in “A” for inhibitor of DNA binding 2 (*ID2*). (C) As in “A” for inhibitor of DNA binding 4 (*ID4*). |

| **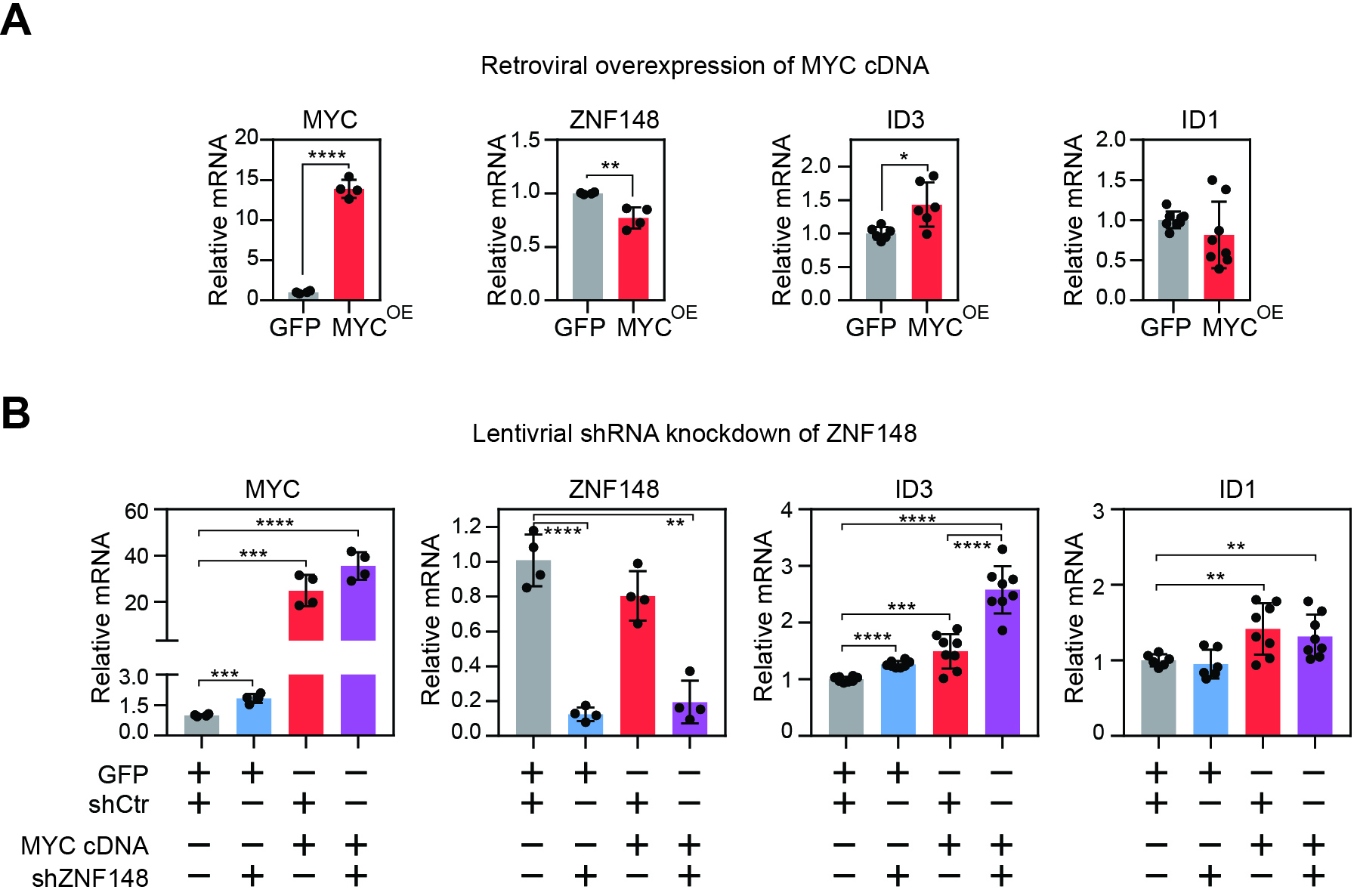** |
| --- |
| **Supplemental Figure 8. MYC-ZNF148-ID1/3 axis in MCF10A cells.** (A) MCF10A cells are transduced with GFP (control) or MYC cDNA (MYC^OE^) expressing retroviruses. RT-qPCR analysis for MYC (n = 4), ZNF148 (n = 4), ID3 (n = 6) and ID1 mRNA (n = 8) transcripts relative to GAPDH and MCF10A-GFP control cells. (B) MCF10A-GFP or MCF10A-MYC cells are transduced with lentiviruses carrying shCtr or shZNF148. RT-qPCR analysis for MYC (n = 4), ZNF148 (n = 4), ID3 (n = 8) and ID1 (n = 8) mRNA transcripts relative to GAPDH and MCF10A-GFP-shCtr control cells. Student's t-test, *P < 0.05. **P <0.01. ***P < 0.001. ****P < 0.0001. Error bars represent ± SD. |
